# A two-microRNA-based signature predicts first-line chemotherapy outcomes in advanced colorectal cancer patients

**DOI:** 10.1038/s41420-018-0133-7

**Published:** 2018-12-18

**Authors:** Jia-huan Lu, Zhi-xiang Zuo, Wei Wang, Qi Zhao, Miao-zhen Qiu, Hui-yan Luo, Zhan-hong Chen, Hai-yu Mo, Feng Wang, Dong-dong Yang, Yun Wang, Xiao-li Wei, Qi-nian Wu, Huai-qiang Ju, Rui-hua Xu, Zhao-lei Zeng

**Affiliations:** 1 State Key Laboratory of Oncology in South China, Collaborative Innovation Center for Cancer Medicine, Sun Yat-sen University Cancer Center, 510060 Guangzhou, China; 2 Foshan First People’s Hospital, 528000 Foshan, China; 3 The Third Affiliated Hospital, Sun Yat-sen University, 510630 Guangzhou, China

## Abstract

Prognostic and predictive markers are needed to predict the clinical outcomes of patients with advanced colorectal cancer (CRC) who receive standard first-line treatments. We performed a prospective cohort study in advanced CRC patients to identify a miRNA signature that could predict the benefit of receiving first-line chemotherapy for these patients. Twenty-one paired tumours and adjacent normal tissues were collected from advanced CRC patients and analysed by miRNA microarrays. Between tumour and normal tissues, 33 miRNAs were differentially expressed and was confirmed by qRT-PCR from another group of 67 patients from a prospective cohort study. A two-miRNA-based signature was obtained using the LASSO Cox regression model based on the association between the expression of each miRNA and the PFS of individual patients. Internal and external validation cohorts, including 40 and 44 patients with advanced CRC, respectively, were performed to prove the prognostic and predictive value of this signature. A signature was built based on two miRNAs, miR-125b-2-3p and miR-933. CRC patients were classified into low- and high-risk groups for disease progression based on this tool. The patients with low risk scores generally had better PFS than those with high risk scores. In the training set, the median PFS in the low- and high-risk groups were 12.00 and 7.40 months, respectively. In the internal validation set, the median PFS in the low- and high-risk groups were 9.90 and 5.10 months, respectively. In the external validation set, the median PFS in the low- and high-risk groups were 9.90 and 6.40 months, respectively. Furthermore, we detected miR-125b-2-3p associated with CRC cell sensitivity to first-line chemotherapy. Our two-miRNA-based signature was a reliable prognostic and predictive tool for tumour progression in patients with advanced CRC, and might be able to predict the benefit of receiving standard first-line chemotherapy in CRC.

## Introduction

Colorectal cancer (CRC) is the third most common cancer diagnosed in both men and women and is the fourth leading cause of cancer-related death worldwide^1,2^. An estimated 1.4 million new cases of CRC are clinically diagnosed globally, resulting in 693,900 patient deaths from CRC in 2012^3^. Over 20% of CRC cases are diagnosed at stage IV, an advanced stage, and higher percentages have been reported in developing countries^4,5^. Chemotherapy is one of the most common treatments for advanced CRC patients. FOLFOX (5-fluorouracil (5-FU), leucovorin and oxaliplatin)/XELOX (capecitabine and oxaliplatin) and FOLFIRI (5-FU, leucovorin and irinotecan) are the first-line chemotherapeutic strategies for advanced CRC^6^. However, these similar chemotherapeutic strategies result in varying responses and different clinical outcomes in advanced CRC^7–9^. Moreover, primary and acquired drug resistance remain significant challenges to achieving satisfactory treatment effects. Thus, identifying effective biomarkers is essential for improving the prediction of treatment responses and guiding treatment decisions in advanced CRC patients.

MicroRNAs (miRNAs) are an abundant class of small non-coding RNAs that typically induce gene silencing or mRNA degradation by binding to the target sites in the 3′ untranslated regions (UTRs) of their targeted mRNAs^10,11^. MiRNAs are involved in varied biological and pathophysiological processes, such as the cell cycle, cell differentiation, proliferation, apoptosis, etc.^12–14^. Apart from their important roles in biological processes, miRNAs also participate in a wide range of diseases, including many types of cancers^15,16^. Accordingly, miRNAs are studied as candidates for diagnostic and prognostic biomarkers and predictors of drug responses^17,18^. Several studies have demonstrated the roles of miRNAs as chemotherapeutic predictors in CRC^19–21^. Compared to a single biomarker, integrating multiple biomarkers into a single model can significantly improve the predictive value^22,23^.

In the present prospective observational study, we aimed to exploit and validate a multiple-miRNA-based signature using the least absolute shrinkage and selection operator (LASSO) Cox regression model to predict the progression-free survival (PFS) and response to controversial chemotherapeutic strategies in advanced CRC patients. We assessed the prognostic and predictive value of this signature in the training set, and then we validated this signature in two validation cohorts. Thus, our study identifies an effective prognostic and predictive biomarker signature for advanced CRC patients receiving standard first-line treatments.

## Results

### Clinicopathological characteristics of patients

A total of 151 patients with histopathologically and clinically diagnosed advanced CRC were enrolled in the present study according to the selection criteria. The patients from SYSUCC were included and randomly assigned to the training set (67 patients) and internal testing set (40 patients), and another 44 patients from FPHFS were recruited as the external validation set. The clinicopathological characteristics of the patients in each set are shown in Table 1.

**Table 1:** Clinicopathologic characteristics of two sets of CRC patients according to the two-miRNAs signature

		Training set (*n* = 67)				Internal testing set (*n* = 40)				Independent validation set (*n* = 44)		
Characteristics	No.	Low risk (%)	High risk (%)	*p* value	No	Low risk (%)	High risk (%)	*p* value	No.	Low risk (%)	High risk (%)	*p* value
Gender				0.68				0.35				0.60
Male	42	18 (42.9)	24 (57.1)		25	17 (68.0)	8 (32.0)		27	18 (66.7)	9 (33.3)	
Female	25	12 (48.0)	13 (52.0)		15	8 (53.3)	7 (46.7)		17	10 (58.8)	7 (41.2)	
Age				0.09				0.62				0.16
<60-year-old	41	15 (36.6)	26 (63.4)		22	13 (68.4)	9 (31.6)		28	20 (71.4)	8 (28.6)	
≥60-year-old	26	15 (57.7)	11 (42.3)		18	12 (66.7)	6 (33.3)		16	8 (50.0)	8 (50.0)	
Tumour location				0.14				0.67				0.72
Colon	50	25 (50.0)	25 (50.0)		25	15 (60.0)	10 (40.0)		29	19 (65.5)	10 (34.5)	
Rectum	17	5 (29.4)	12 (70.6)		15	10 (66.7)	5 (33.3)		15	9 (66.7)	6 (33.3)	
Tumour grade				0.35				0.55				0.70
Low	6	1 (16.7)	5 (83.3)		3	1 (33.3)	2 (66.7)		5	4 (80.0)	1 (20.0)	
Median	57	27 (47.4)	30 (52.6)		34	22 (64.7)	12 (35.3)		38	23 (60.5)	15 (39.5)	
High	4	2 (50.0)	2 (50.0)		3	2 (66.7)	1 (33.3)		1	1 (100.0)	0 (0.0)	
Metastatic location				0.68				0.57				0.63
Liver	42	18 (42.9)	24 (57.1)		30	18 (60.0)	12 (40.0)		34	21 (61.8)	13 (38.2)	
Others	25	12 (48.0)	13 (52.0)		10	7 (70.0)	3 (30.0)		10	7 (70.0)	3 (30.0)	
Metastatic type				0.82				0.43				0.91
Synchronous	50	22 (44.0)	28 (56.0)		27	18 (66.7)	9 (33.3)		27	17 (63.0)	10 (27.0)	
Metachronous	17	8 (47.1)	9 (52.9)		13	7 (53.8)	6 (46.2)		17	11 (64.7)	6 (35.3)	
Chemotherapy strategy				0.69				0.28				0.91
FOLFOX	55	24 (43.6)	31 (56.4)		31	18 (58.0)	13 (42.0)		27	17 (63.0)	10 (27.0)	
FOLFIRI	12	6 (50.0)	6 (50.0)		9	7 (77.8)	2 (22.2)		17	11 (64.7)	6 (35.3)	
Response for chemotherapy				<0.001				<0.001				<0.001
CR + PR	23	15 (65.2)	8 (34.8)		10	9 (90.0)	1 (10.0)		11	6 (54.5)	5 (45.5)	
SD	31	11 (35.5)	20 (64.5)		21	13 (61.9)	8 (38.1)		21	17 (81.0)	4 (19.0)	
PD	13	4 (30.8)	9 (69.2)		9	3 (33.3)	6 (66.7)		12	5 (41.7)	7 (58.3)	

### Building a two-miRNA-based prognostic classifier with an integrated marker selection approach and Cox regression

**Fig. 1:**
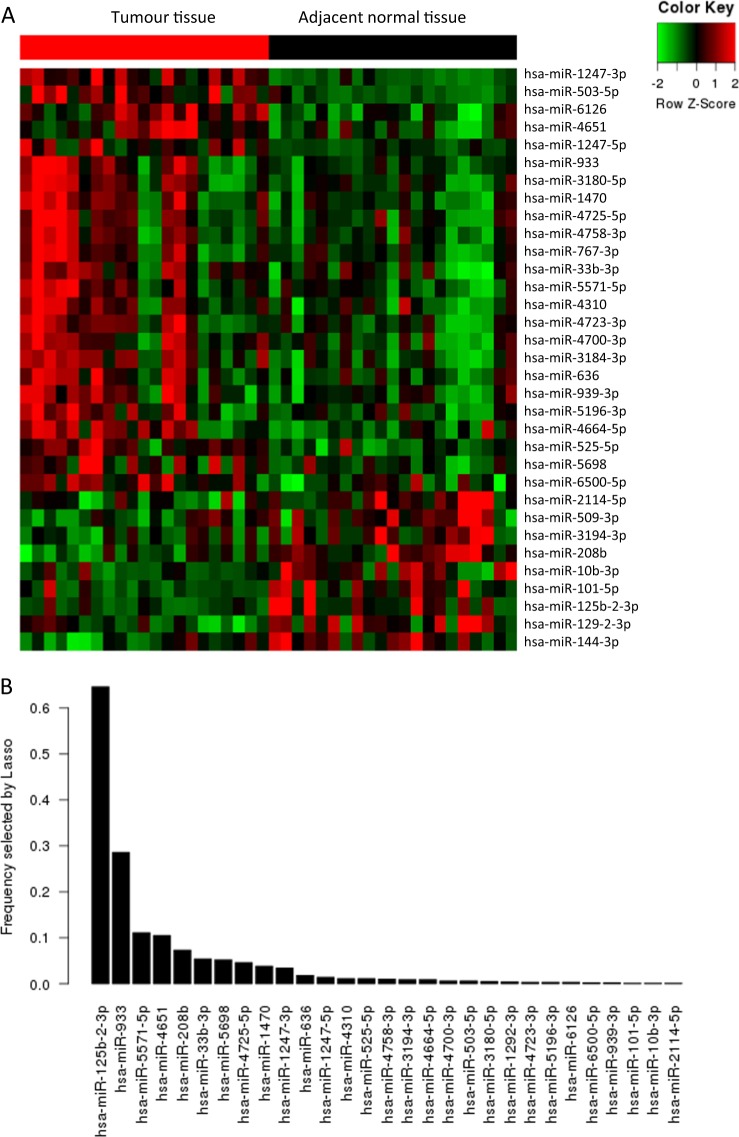
Construction of the two-miRNA-based signature. **a** Hierarchical clustering of 21 paired tumour tissues and adjacent normal mucosa with the 33 differentially expressed miRNAs using Euclidean distance and average linkage clustering. **b** RMIP for each of the 33 differentially expressed miRNAs (also explained by an observed frequency in 1000 resamples) was measured by LASSO Cox regression analysis

Twenty-one pairs of tumour and adjacent normal tissues from advanced CRC patients were used in the microarray analysis. To obtain the most significant miRNAs for classifying high- and low-risk progressive patients, we applied an integrated marker selection method, consisting of differential expression analysis, uni-variable Cox regression analysis and LASSO bagging variable selection. The flowchart of microRNA filtration is illustrated in Supplementary Fig. S1. First, using the microarray data matrix, differential expression analysis was performed with the limma package between tumour and normal tissues^24^. A total of 178 upregulated miRNAs and 231 down-regulated miRNAs were identified in the tumour samples. Then, with the same data set, Cox regression analyses with a Wald threshold of *p* < 0.1 were run individually to screen the progression-related markers. A total of 33 miRNAs were identified as differentially expressed between the tumour and adjacent normal samples and were correlated with the patients’ PFS (*p* < 0.1, Kaplan–Meier method). Hierarchical clustering showed that the 21 pairs of tumour and adjacent normal tissues were separated into two discrete groups (Fig. 1a). The expression of these 33 miRNAs in tumour and adjacent normal tissues was validated by qRT-PCR (Supplementary Fig. S2), confirming the differential expression of these miRNAs between tumour and adjacent normal tissues.

To further narrow down a miRNA-based signature for patients with advanced CRC, we sequentially applied a LASSO bagging method on the qRT-PCR dataset from the training set. Briefly, the LASSO method is commonly used for regression with high-dimensional predictors, and its extended model has been widely applied to the Cox proportional hazard regression model for the analysis of high-dimensional survival data^25^. For LASSO bagging, bootstrap samples of the data (sampling with replacement with a sample size equal to that of the original data) were used to perform the entire LASSO Cox procedure, including optimal tuning parameter selection (also called λ) and variable selection steps. In the present analysis, we obtained 1000 resamples and used different optimal λ parameters selected via 1-SE (standard error) criteria for each resample. We then calculated the RMIP for each miRNA. The RMIP is a measure of how likely a given miRNA is selected by the LASSO procedure if the dataset is perturbed. We observed continuing sharp decreases of RMIP until the third miRNA miR-5571-5p as ordered by the RMIP of each miRNA. As a result, two miRNAs, miR-125b-2-3p and miR-933, were selected for building a prognostic classifier (Fig. 1b). Additionally, a two-variable Cox regression analysis was performed on the same data to determine the coefficients of the two miRNAs, which were −0.259 for miR-125b-2-3p and 0.092 for miR-933.

Using a two-variable Cox regression model, we obtained the risk score for each patient (risk score = ${\mathrm{0}}{\rm{.092 \times miR - 933}} - {\mathrm{0}}{\mathrm{.259 \times }}\;{\rm{miR - 125b - 2 - 3p}}$). Based on the signature, the risk score for each patient in the training cohort was determined. The median value of the risk score in the training set was taken as the cut-off value (0.022), and all the patients were then assigned to low- or high-risk groups based on their risk scores.

Post hoc statistical power analysis was used to assess adequacy of research design. The post hoc statistical power analysis showed an adequate power of 88.3% in training cohort and 98.4% for all samples.

### Prognostic value of the miRNA signature

**Fig. 2:**
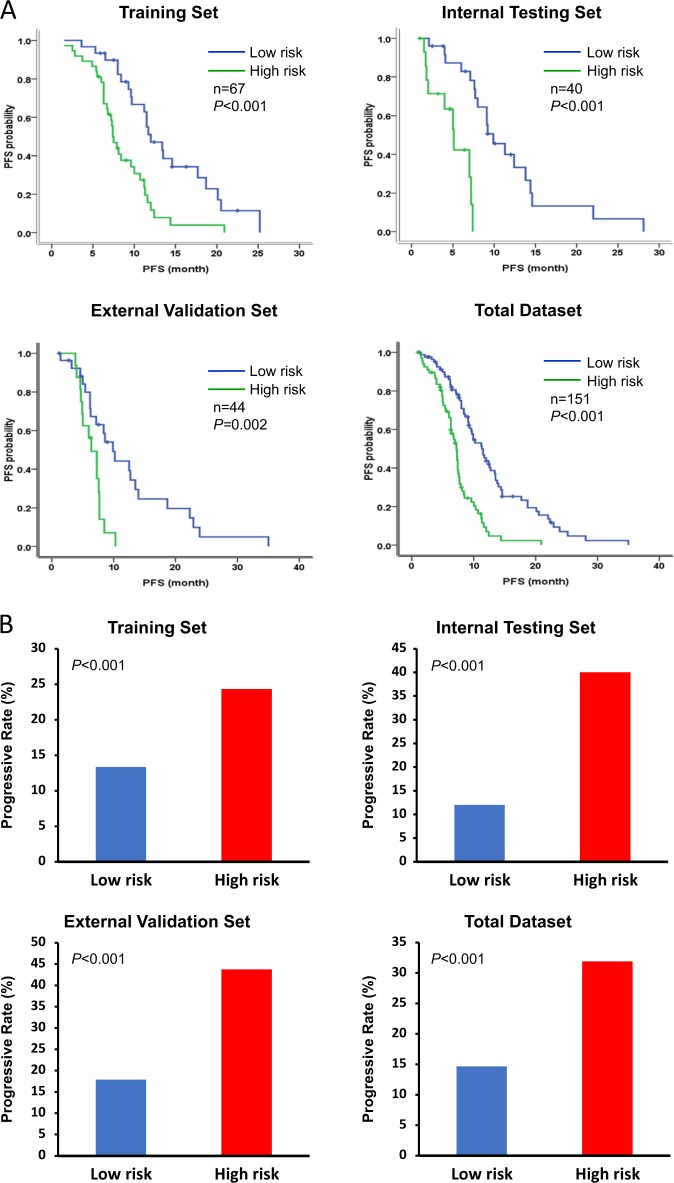
Distribution of the risk score and response status to first-line chemotherapy in three cohorts and total dataset. **a** Kaplan–Meier survival analysis for the patients with advanced CRC according to the two-miRNA-based signature in the training set, internal testing set, external validation set and total dataset. **b** Progressive rate between the low-risk and high-risk groups in three cohorts and all dataset

To examine whether the signature was associated with PFS, we applied multivariate Cox proportional hazards regression analyses in the training, internal testing and external validation sets (Table 2). The patients with high risk scores generally displayed worse PFS than those with lower risk scores in the training set; the median PFS was 12.00 months (95% CI 9.89–14.11 m) for the low-risk group and 7.40 months (95% CI 6.56–8.24 m) for the high-risk group (*p* < 0.001). To confirm the prognostic accuracy of the two-miRNA-based signature, we conducted analyses using the internal testing and external validation sets. Similar results were observed in these two sets. In the internal testing set, the median PFS was 9.90 months (95% CI 6.89–12.91 m) for the low-risk group and 5.10 months (95% CI 3.57–6.63 m) for the high-risk group (*p* < 0.001). In the external validation set, the median PFS was 9.90 (95% CI 7.33–12.47 m) for the low-risk group and 6.40 months (95% CI 4.87–7.93 m) for the high-risk group (*p* = 0.002). For all the patients, the median PFS in the low- and high-risk groups was 11.30 (95% CI 9.36–13.24 m) and 7.20 months (95% CI 6.69–7.81 m), respectively (*p* < 0.001) (Fig. 2a). The progressive rate was 13.33% (4/30) for the low-risk group and 24.32% (9/37) for the high-risk one in the training set (*p* < 0.001); 12.00% (3/25) for the low-risk group and 40.00% (6/15) for the high-risk group in the internal testing set (*p* < 0.001); and 17.86% (5/28) vs. 43.75% (7/16), respectively, in the external validation set (*p* < 0.001). For all patients, the progressive rate was 14.63% (12/82) for the low-risk group and 31.88% (22/69) for the high-risk one, respectively (*p* < 0.001; Fig. 2b).

**Table 2:** Multivariate Cox proportional hazards regression analysis of the clinicopathologic characteristics and integrated microRNA signature with PFS

	Training set (*n* = 67)		Internal testing set (*n* = 40)		Independent validation set (*n* = 44)	
	HR (95% CI)	*p*	HR (95% CI)	*p*	HR (95%CI)	*p*
Gender	1.034 (0.584–1.830)	0.910	1.410 (0.661–3.007)	0.374	0.898 (0.456–1.769)	0.757
Age (<60-year-old vs ≥ 60 year-old)	0.771 (0.441–1.347)	0.361	0.667 (0.269–1.657)	0.383	0.726 (0.370–1.426)	0.353
Tumour location (colon vs rectum)	1.185 (0.651–2.160)	0.578	1.084 (0.505–2.325)	0.836	1.388 (0.669–2.880)	0.379
Tumour grade (low vs median/high)	0.531 (0.232–1.215)	0.134	2.743 (1.020–7.383)	0.046	1.576 (0.475–5.229)	0.457
Metastatic location (without vs with liver metastasis)	1.004 (0.569–1.770)	0.989	2.446 (0.908–6.587)	0.077	2.086 (0.842–5.171)	0.112
Metastatic site number (one vs more than one)	1.601 (1.119–2.290)	0.010	1.192 (0.792–1.792)	0.400	1.853 (1.070–3.210)	0.028
Metastatic type (synchronous vs metachronous)	0.617 (0.315–1.209)	0.159	1.099 (0.481–2.512)	0.823	1.235 (0.624–2.444)	0.544
Chemotherapy strategy (FOLFOX vs FOLFIRI)	1.151 (0.577–2.299)	0.690	0.756 (0.304–1.877)	0.546	0.741 (0.372–1.478)	0.395
Response for chemotherapy (CR + PR + SD vs PD)	4.608 (2.337–9.083)	<0.001	12.033 (4.008–36.130)	<0.001	2.802 (1.276–6.152)	0.010
miR-125b-2-3p (low vs high expression)	0.526 (0.29–0.952)	0.034	0.443 (0.203–0.969)	0.041	0.487 (0.244–0.971)	0.041
miR-933 (low vs high expression)	0.875 (0.490–1.565)	0.653	0.834 (0.434–1.941)	0.340	0.616 (0.309–1.229)	0.169
miRNA signature (low vs high risk)	2.817 (1.584–5.009)	<0.001	7.797 (2.525–24.089)	<0.001	3.153 (1.444–6.887)	0.004

**Fig. 3:**
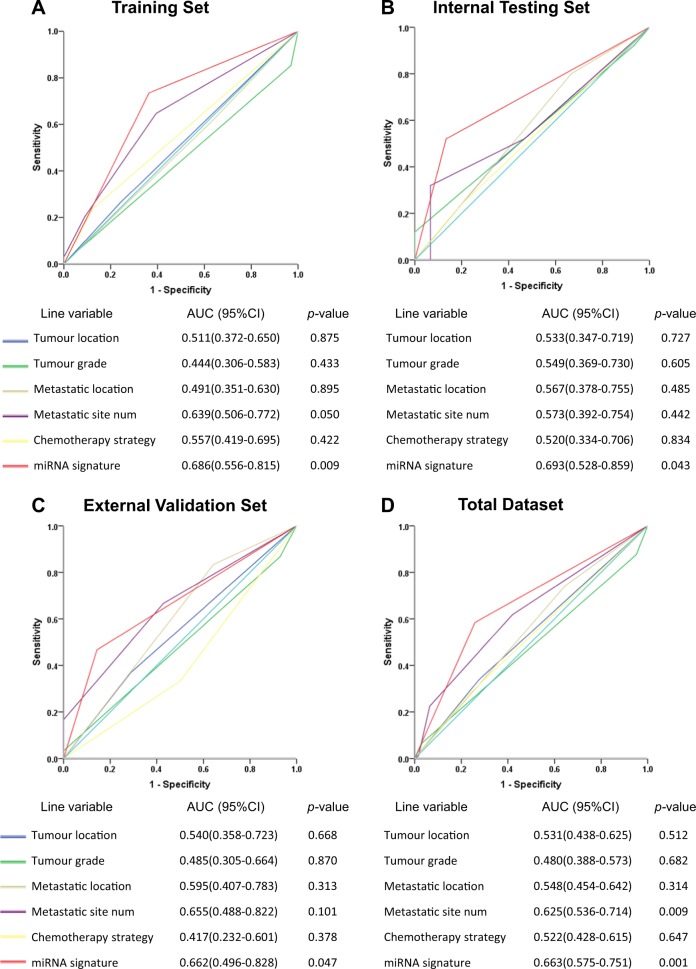
Time-dependent receiver-operating characteristic (ROC) curves for advanced CRC patients comparing the prognostic accuracy for first-line chemotherapy response by tumour location (colon vs rectum), tumour grade (low vs median/high), metastatic location (without vs with liver metastasis), metastatic site number (1 vs ≥2), chemotherapy strategy (FOLFOX vs FOLFIRI) and the two-miRNA-based signature (high risk vs low risk) in the **a** training set, **b** internal testing set, **c** external validation set and **d** total dataset. Area under curve (AUC) was calculated and its 95% CI was estimated by SPSS

To further assess the prognostic value of the two-miRNA signature on risk score prediction, we calculated the time-dependent receiver operating characteristic (ROC) in the three cohorts respectively. The two-miRNA-based signature showed significantly higher prognostic value than any other single clinicopathological risk factor (*p* < 0.05) (Fig. 3).

### Predictive value of the signature for chemotherapy sensitivity

We used multivariate Cox proportional hazards regression analysis to test the predictive value of the signature for first-line chemotherapy sensitivity in advanced CRC cases (Table 3). The interactions between the risk status and patient response to chemotherapy were analysed. The interactions between these were dramatically significant in the training set (HR 3.644; 95% CI 1.528–8.690; *p* = 0.004), in the internal testing set (HR 8.426; 95% CI 2.592–27.390; *p* < 0.0001), and in the external validation set (HR 2.653; 95% CI 1.031–6.829; *p* = 0.043). These data suggested that this two-miRNA-based signature might be valuable for predicting the benefit of standard first-line chemotherapy in advanced CRC.

**Table 3:** Interaction analysis of the signature and chemotherapy in relationship with PFS using multivariate Cox proportional hazards regression

	Training set (*n* = 67)		Internal testing set (*n* = 40)		Independent validation set (*n* = 44)	
	HR (95% CI)	*p*	HR (95% CI)	*p*	HR (95% CI)	*p*
Response to chemotherapy (CR + PR + SD vs PD)	3.103 (1.436–6.708)	0.004	10.263 (3.463–30.413)	<0.001	2.802 (1.276–6.152)	0.010
miRNA signature (low vs high risk)	2.817 (1.584–5.009)	< 0.001	7.797 (2.525–24.089)	<0.001	3.153 (1.444–6.887)	0.004
Interaction	3.644 (1.528–8.690)	0.004	8.426 (2.592–27.390)	<0.001	2.653 (1.031–6.829)	0.043

### Drug effects of the two microRNAs

**Fig. 4:**
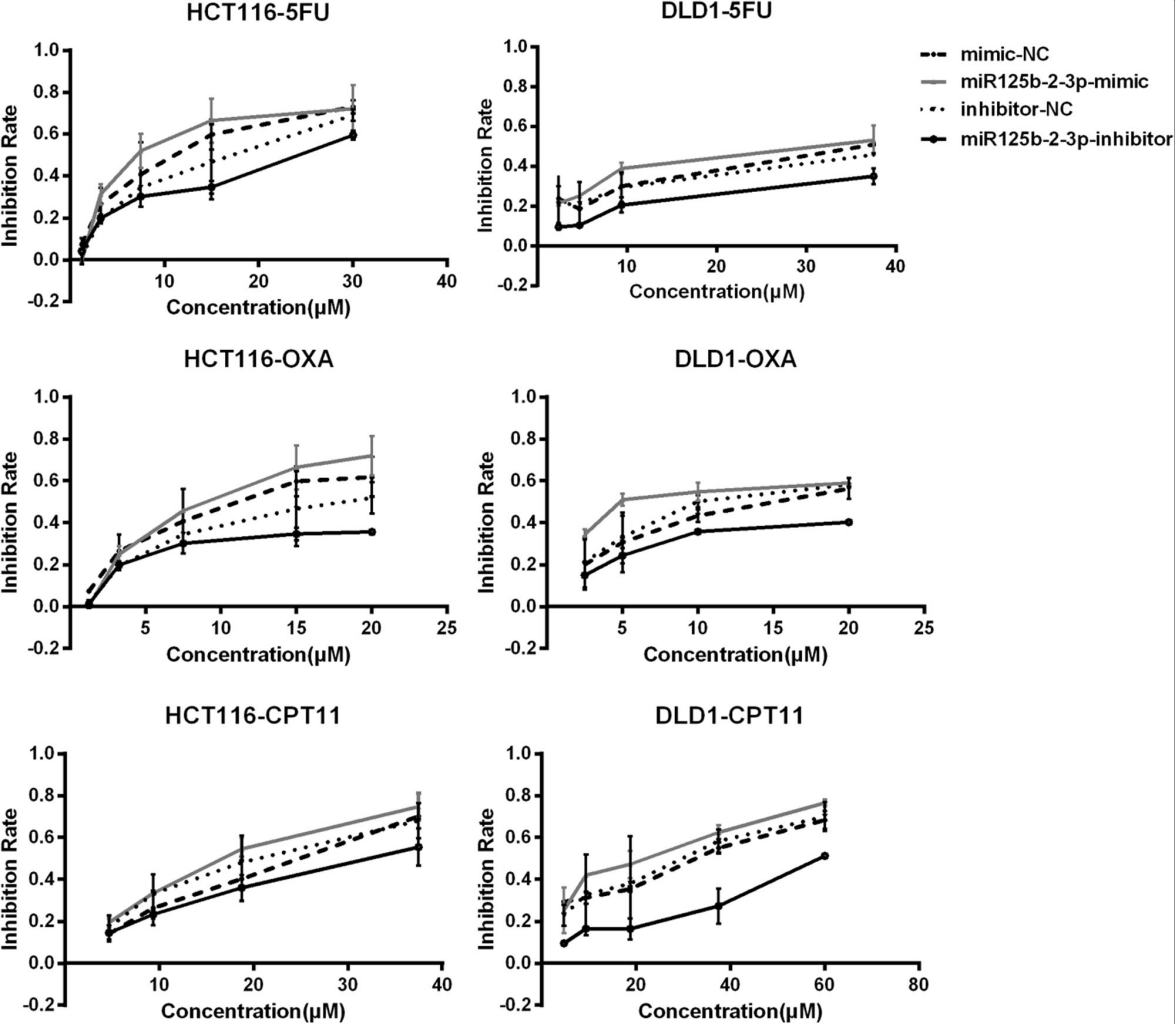
Effects of miRNA-125b-2-3p in HCT-116 and DLD-1 cells treated with 5-FU, oxaliplatin and CPT-11. The cells were treated with specific mimics and inhibitors, and the inhibition rates were measured by MTS assay after anticancer drug treatment for 72 h. Points, mean (*n* = 3); bars, SD

To explore the effect of miR-125b-2-3p and miR-933 on the activity of anticancer drugs, the MTS assay was performed in the CRC cell lines HCT116 and DLD-1. For each miRNA, specific mimics and inhibitors were used for functional experiments. The half maximal inhibitory concentrations (IC50) of anticancer drugs were calculated. As shown in Fig. 4 and Table 4, the cells with knockdown of miR-125b-2-3p were more resistant to the anticancer drugs fluorouracil, oxaliplatin and CPT-11. In addition, the cells with overexpression of miR-125b-2-3p were more sensitive to all three anticancer drugs. However, no significant change due to the anticancer drugs was found in the miR-933 knockdown or overexpression cell lines.

**Table 4:** The IC50 of anticancer drugs in CRC cell lines

	5FU	Oxaliplatin	CPT11
HCT116 cell line			
mimic-NC	9.45 ± 1.86	11.49 ± 1.36	20.81 ± 3.18
miR933-mimic	4.82 ± 1.09	6.77 ± 0.74	5.39 ± 0.28
miR125b-2-3p-mimic	6.27 ± 0.58	9.69 ± 0.25	17.58 ± 0.59
inhibitor-NC	14.48 ± 3.23	18.70 ± 1.63	18.37 ± 0.25
miR933-inhibitor	19.70 ± 3.18	26.48 ± 1.50	30.26 ± 11.96
miR125b-2-3p-inhibitor	25.79 ± 1.60	29.10 ± 0.63	30.92 ± 4.40
DLD-1 cell line			
mimic-NC	26.04 ± 10.09	13.29 ± 1.29	25.09 ± 5.51
miR933-mimic	18.59 ± 8.00	7.79 ± 1.16	20.28 ± 1.39
miR125b-2-3p-mimic	18.41 ± 1.85	9.07 ± 1.60	20.31 ± 0.42
inhibitor-NC	27.07 ± 1.46	15.82 ± 1.03	29.04 ± 2.28
miR933-inhibitor	65.49 ± 7.57	32.72 ± 1.66	48.41 ± 1.95
miR125b-2-3p-inhibitor	71.76 ± 3.99	50.42 ± 9.81	60.97 ± 9.56

## Discussion

Traditional clinicopathologic characteristics, such as gender, age, tumour size, tumour grade, and lymph node status, are rarely associated with the clinical response to chemotherapy of advanced CRC patients. However, the prediction of clinical response in metastatic CRC is vital for guiding therapeutic decisions. In the present study, we developed and verified a novel two-miRNA-based classifier to predict the disease progression and the benefit of receiving standard first-line chemotherapy in advanced CRC. Our results showed that this classifier could effectively assign advanced CRC patients into high- or low-risk groups with significant differences in PFS and that patients categorized as high risk based on the signature had worse survival outcomes after chemotherapy. Further use of this classifier might help identify advanced CRC patients who are most likely to benefit from first-line chemotherapy. Thus, the present miRNA signature for advanced CRC patients is a valuable prognostic and predictive tool, indicating that patients who are classified as low risk based on their risk scores have much lower chance of disease progression and have better responses to chemotherapy.

There were plenty of miRNAs which were identified differentially expressed in CRC compared with normal tissues. Among these miRNAs, two miRNAs enrolled in the signature, miR-125b and miR-933, have previously been reported as playing potential roles in cancer. MiR-125b acts as either an oncogene or tumour suppressor and shows heterogenic expression in different carcinomas. For example, miR-125b is down-regulated in ovarian, bladder and breast cancer, which suggests a tumour suppressor function^26–28^. In contrast, miR-125b is upregulated in leukaemia and prostate cancer, where this molecule seems to act as an oncogene^29,30^. These contradictory findings also occurred in CRC. On the one hand, downregulation of miR-125b was associated with lymph node metastasis in CRC, as shown by significantly increased cell invasion, migration, and MMP activity^31^. On the other hand, high levels of miR-125b expression in CRC were associated with poor survival^32^. In the present study, miR-125b-2-3p was down-regulated in CRC tumour tissues, and the expression of this molecule was positively correlated with PFS, which was consistent with the previous metastatic CRC study^31^.

Moreover, our data showed that miR-125b-2-3p affected CRC cell sensitivity to chemotherapeutic therapy. The effects of miR-125b-2-3p knockdown were more notable than those of its overexpression in CRC cells since the expression of miR-125b-2-3p was high in these two CRC cell lines. On the other hand, as we known, stromal cells play important roles in CRC biology and affect the response to chemotherapy. It was reported that the expression of miR-125b-2-3p in CRC stroma has no significant difference with that in the epithelia^33^. However, there was no study to compare the expression of miR-125b-2-3p in stromal cells and in cancer cells. Due to lack of microdissection procedure, our study cannot exclude the effect of stromal cells in CRC, so further study is needed to explore the role of miR-125b-2-3p in stromal cells.

In our study, the miR-933 knockdown or overexpression cells had no significant change in their response to the anticancer drugs. Furthermore, the Cox regression analysis showed that there was no significant association between the expression of miR-933 and CRC patients’ PFS, which might mean that the miR-933 is not an independent prognostic and predictive biomarker in CRC.

The function and mechanism of microRNA-933 in CRC tumourigenesis, progression, and response to chemotherapy has not been thoroughly investigated. However, several studies exploring single-nucleotide polymorphisms in miR-933 have shown that this molecule is associated with susceptibility to several types of human cancers^34–36^, although further mechanistic exploration is needed.

However, our study was a prospective observational study and not a clinical trial; therefore, there may be some inherent biases of such a study format. Moreover, the patients enrolled in the present study were all Chinese individuals whose clinical characteristics might be different from those of other ethnic groups; thus, the generalizability of this study might be limited. Clearly, an open-label, multicentre, randomized clinical trial is needed to further validate this novel signature. Furthermore, the mechanism for how miR-125b-2-3p affects CRC sensitivity to chemotherapy warrants further exploration.

In summary, the present study shows that the two-miRNA signature can successfully classify advanced CRC patients into groups with high and low risks of tumour progression. In addition, this signature might be a reliable prognostic and predictive tool for tumour progression in patients with advanced CRC and might be able to predict the benefit of receiving standard first-line chemotherapy in CRC.

## Materials and methods

### Patients and clinical database

This prospective observational study was started in 2008. A total of 151 patients with histopathologically and clinically diagnosed advanced CRC from 1 January 2008 to 30 June 2014 were enrolled in this project. The following were the criteria for enrolment: (1) patients diagnosed with advanced CRC; (2) histologically confirmed CRC with at least one measurable lesion as defined by the Response Evaluation Criteria in Solid Tumours; (3) patients who received National Comprehensive Cancer Network (NCCN)-guided first-line chemotherapy (FOLFOX or FOLFIRI strategy); (4) patients with completed clinicopathological information and follow-up data; (5) patients have not received previous chemotherapy for the target lesions, however, patients who had received adjuvant/neoadjuvant chemotherapy were enrolled if the interval was more than 12 months after the end of chemotherapy; and (6) sufficient formalin-fixed paraffin-embedded (FFPE) tissues for further study. Prior to primary chemotherapy, tumour samples were acquired during palliative operations or colonoscopy biopsies and embedded in paraffin.

For the training and internal testing sets, 107 metastasis CRC patients treated at Sun Yat-sen University Cancer Center (SYSUCC, Guangzhou, China) were included from 1 January 2008 to 30 June 2014. These 107 patients were randomly divided into training and internal testing sets (67 and 40 patients, respectively). To verify the prognostic or predictive accuracy of the signature, we included another 44 advanced CRC patients as the independent external validation cohort from the Foshan First People’s Hospital (FFPH), Foshan, between 1 December 2008 to 31 December 2013. The same inclusion and exclusion criteria were used. Follow-up procedure for these patients was finished on 31 July 2016. The median follow-up time was 44.6 month. Additionally, to obtain the miRNA expression profiles, 21 frozen tumour and paired adjacent normal mucosa samples from metastatic CRC patients obtained at SYSUCC from 1 January 2003 to 31 December 2005 were used.

The clinical and clinicopathological classification and stage were clarified according to the American Joint Committee on Cancer (AJCC) criteria. Prior to carrying out the research, this project was approved by the Institutional Research Ethics Committee and all the participants signed informed consent forms. All the patients received tumour assessments every 6 weeks during therapy and at the end of treatment. PFS was defined as the period between the initiation treatment of first-line chemotherapy and the date of disease progression or death. The blinded endpoint study was not applicable in this project. The study was followed REMARK guideline (supplementary Table 1).

### Sample preparation

The FFPE tissue samples were composed of at least 80% tumour cells. Total RNA was isolated from 151 metastatic CRC samples and 21 paired CRC tumour and adjacent normal tissues as previously described^37^. The purity and concentration of RNA were quantified by using a Nanodrop 2000 spectrophotometer (Thermo Fisher Scientific, USA).

RNA labelling, hybridization and array scanning were carried out as previously described and performed by CapitalBio (Capital-Bio Corp., Beijing, China)^38^. Briefly, total RNA was purified and concentrated using the mirVana™ miRNA Isolation Kit (Ambion, Austin, TX, USA). Purified RNA was labelled with fluorescein Cy3 and hybridization was conducted using an Agilent Human miRNA Microarray (miRBase release 19.0). The fluorescence intensities were converted into digital data and Log2 transformed using Feature Extraction (version 10.7). Differential gene expression was analysed by using GeneSpring software version 12.0 (Agilent, USA). The miRNAs were categorized as significantly differentially expressed if the *p* value was lower than 0.05. Heat maps were formed using the Cluster 3.0 package software. Microarray data were deposited into the Gene Expression Omnibus (GEO) database of the National Center for Biotechnology Information (NCBI) (accession no. GSE108153).

MiRNA expression was detected by qRT-PCR in the 151 CRC samples and 21 paired CRC and adjacent normal tissues. Total RNA was obtained with TRIzol reagent (Life Technologies, Carlsbad, California, USA), and 2 μg total RNA was reverse transcribed using the All-in-One™ miRNA First-Strand cDNA Synthesis Kit (GeneCopoeia Inc., USA). The All-in-One™ miRNA qPCR Kit (GeneCopoeia Inc., USA) and Roche Lightcycler 480 instrument (Roche Diagnostics, Basel, Switzerland) were used for qRT-PCR analysis. All experiments were conducted according to the manufacturer’s instructions. The results were normalized to U48 expression and analysed using in the 2-Δct method. The primers were synthesized by GeneCopoeia, Inc.

### Identification of candidate miRNAs and development of the miRNA signature

We performed the miRNA microarray on 21 paired CRC cancer and adjacent normal frozen tissues samples. We identified the differentially expressed miRNAs using the significance analysis of microarrays with a false discovery rate (FDR) < 0.05. By combining the RNA expression data from the microarray and follow-up data, we obtained 33 significantly differentially expressed miRNA candidates correlated with PFS by Cox regression analysis (*p* < 0.1).

Differential miRNA expression was examined by qRT-PCR in the training set and Cox proportional hazard regression modelling was then performed to analyse the correlation between RNA expression and PFS data in patients. The miRNA signature was validated in the internal and external validation sets. The prognostic or predictive accuracy of each feature and the miRNA-based signature was investigated by time-dependent receiver operating characteristic (ROC) analysis, which was measured by the area under the curve.

### LASSO bagging variable selection

To determine the miRNAs most predictive of a high/low recurrence risk signature, we utilized the LASSO bagging strategy. At the data preparation step, we first obtained 33 candidate markers from the differential expression and uni-variable Cox regression analysis described above, and we then examined their RNA abundance in a training data set of 67 cancer samples using RT-PCR. Since the relative RT-PCR values of miRNA in each sample were too small to manage, we applied logarithmic transformation (2 bases) on 10,000 multiplied matrices to normalize the values into a readable range. As a result, a matrix of 33 variables and 67 data points (samples) was built for further LASSO bagging analysis, the procedure for which is described as follows: (1) the data points were resampled 1000 times with replacement to generate 1000 training matrices. (2) For each matrix with recurrent survival outcomes, we performed LASSO Cox regression analysis using 10-fold cross validation. The tuning parameter λ was selected by 1-SE (standard error), and we finally obtained a list of variables that had beta-coefficients different than zero in LASSO. (3) All variable lists obtained in each resample were combined to calculate the resample model inclusion proportion (RMIP) for each miRNA (also explained by an observed frequency in 1000 resamples). (4) The RMIP was used as the weight of each variable, and the top two miRNAs were selected as candidate markers for building the signature. All running analysis scripts were programmed with R (v3.3.1), and LASSO was adopted from the glmnet package (2.0-10).

### Cell culture

The HCT116 and DLD1 CRC cell lines were purchased from ATCC (Manassas, VA, USA) and cultured in RPMI1640 basic medium (1 × ) with 10% foetal bovine serum (Thermo Fisher Scientific, Carlsbad, California, USA) at 37 °C with 5% CO_2_. All cell lines were tested and authenticated by short tandem repeat DNA testing at Beijing Microread Gene Tech. Co., Ltd. (Beijing, China) prior to use.

### MTS assay

Cells were seeded onto 96-well plates at a density of 3 × 10^3^ cells/well and incubated with irinotecan, fluorouracil and oxaliplatin (Selleck, Houston, TX, USA) for 72 h. Then, the cells were stained with 20 μl of sterile MTS (3-(4, 5-dimethylthiazol-2-yl) -5- (3-carboxymethoxyphenyl) -2- (4-sulfophenyl)-2H-tetrazolium) dye (Promega, Madison, WI, USA) for 2 h at 37 °C. The absorbance was measured at a wavelength of 490 nm. All experiments were conducted in triplicate.

### Statistical analysis

Student’s *t* test was used to compare continuous variables between paired tumour and adjacent normal tissues. For survival analyses, the curves were plotted with the Kaplan-Meier method, and the different groups were compared using the log-rank test. Adjusted hazard ratios (HRs) with 95% confidence intervals (CI) were calculated using Cox proportional hazards modelling. The median value of the risk score in the training set was taken as the cut-off value. Post hoc statistical power analysis was calculated to evaluate the study sample size. All statistical tests were two-sided and considered significant at two-sided *p* values < 0.05, unless specifically stated. All the data analyses above were performed using SPSS version 22.0 statistical software or R3.1.2 (R Foundation for Statistical Computing) software.

## Supplementary information

Supplementary Figure Legends

Supplemental Figure S1

Supplemental Figure S2

Supplemental Table 1

Supplemental Table 2
