# Supplementary material for: A two-microRNA-based signature predicts first-line chemotherapy outcomes in advanced colorectal cancer patients
Source: Cell Death Discov. 2018 Dec 18;4:116. doi: 10.1038/s41420-018-0133-7 (PMC6299080; doi:10.1038/s41420-018-0133-7)
Supplement: Supplementary file 1 — Supplementary Figure Legends [file 41420_2018_133_MOESM1_ESM.docx]

Supplementary Fig S1. The flowchart of building a two-miRNA-based prognostic classifier with an integrated marker selection approach and Cox regression.

Supplementary Fig S2. Validation of the expression of each mRNA incorporated in the integrated signature in the 21 paired tumour and adjacent normal tissues from the training set using qRT-PCR. Expression levels of these mRNAs measured by qRT-PCR was notably different between the tumour and non-cancer breast tissues and were significantly correlated with their microarray data.
