# Supplementary figures and images for: A two-microRNA-based signature predicts first-line chemotherapy outcomes in advanced colorectal cancer patients
Source: Cell Death Discov. 2018 Dec 18;4:116. doi: 10.1038/s41420-018-0133-7 (PMC6299080; doi:10.1038/s41420-018-0133-7)

Suppl. Fig. 1

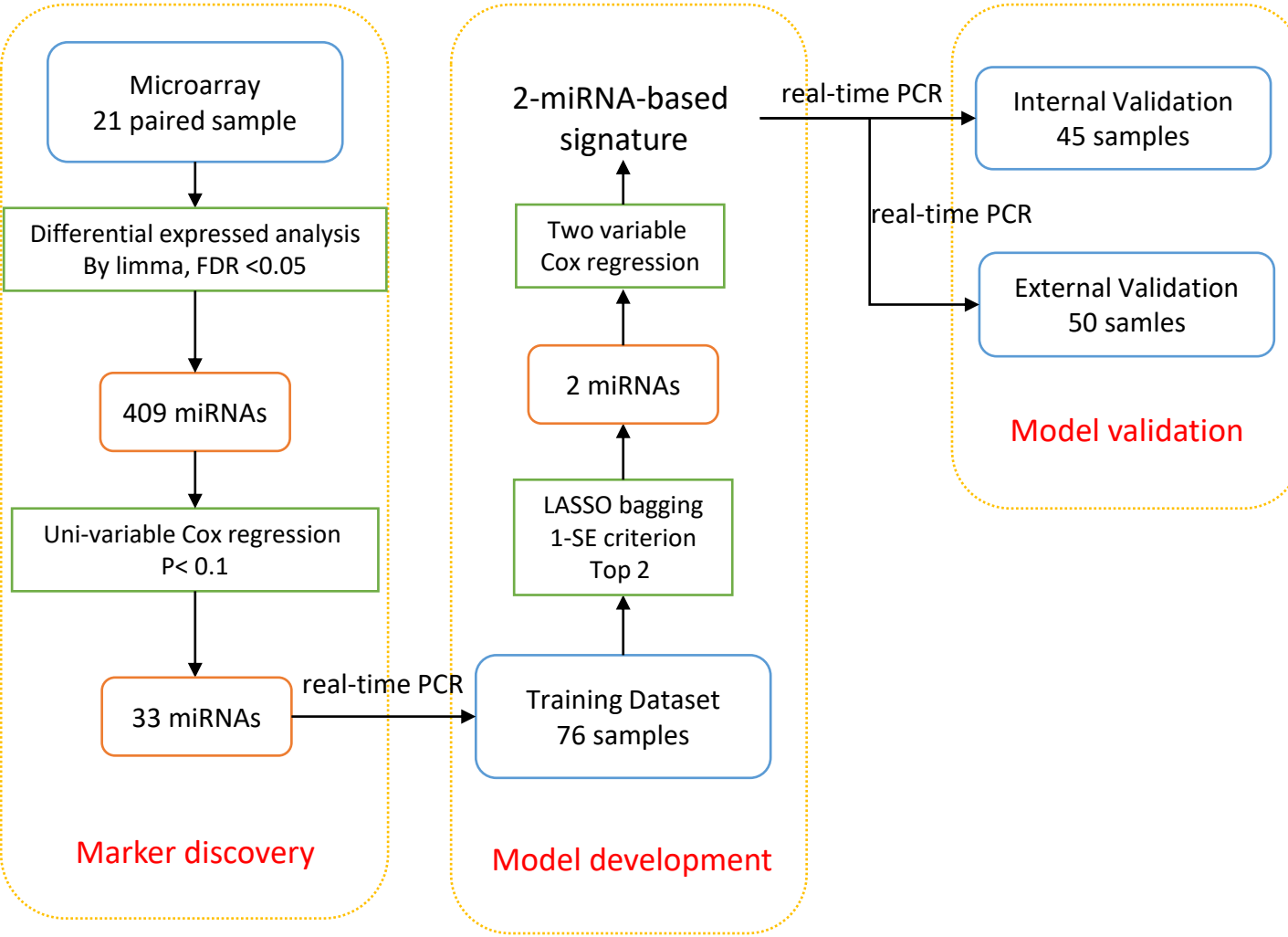

Supplement: Supplementary file 2 — Supplemental Figure S1 [file 41420_2018_133_MOESM2_ESM.pdf]

Suppl. Fig. 2

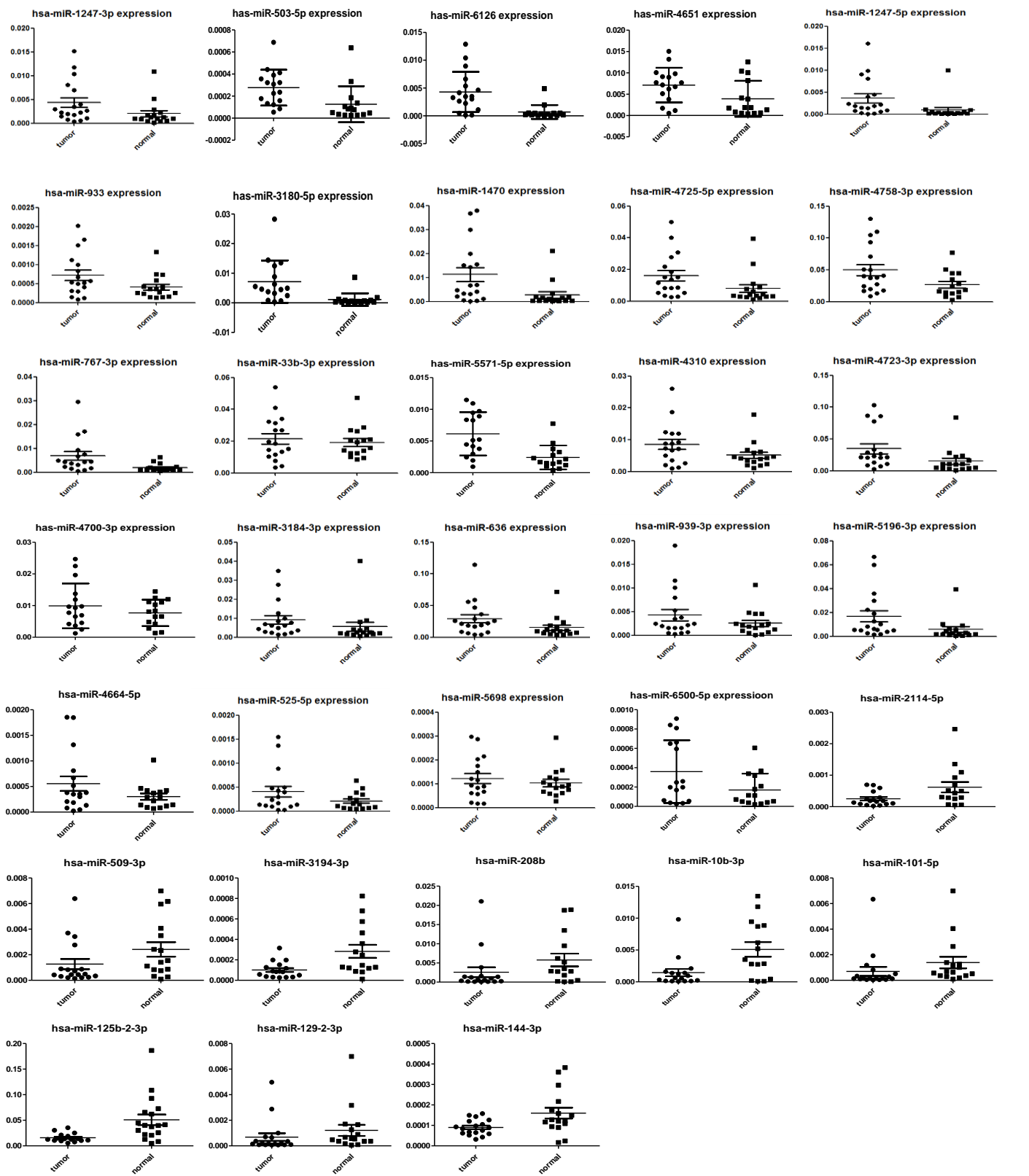

Supplement: Supplementary file 3 — Supplemental Figure S2 [file 41420_2018_133_MOESM3_ESM.pdf]
