# Supplementary material for: A two-microRNA-based signature predicts first-line chemotherapy outcomes in advanced colorectal cancer patients
Source: Cell Death Discov. 2018 Dec 18;4:116. doi: 10.1038/s41420-018-0133-7 (PMC6299080; doi:10.1038/s41420-018-0133-7)
Supplement: Supplementary file 5 — Supplemental Table 2 [file 41420_2018_133_MOESM5_ESM.pdf]

REMARK table involved in this study

Introduction

In the present prospective observational study, we aimed to exploit and validate a multiple-miRNA-based signature using the least absolute shrinkage and selection operator (LASSO) Cox regression model to predict the progression-free survival (PFS) and response to controversial chemotherapeutic strategies in advanced CRC patients. We assessed the prognostic and predictive value of this signature in the training set, and then we validated this signature in two validation cohorts. Thus, our study identifies an effective prognostic and predictive biomarker signature for advanced CRC patients receiving standard first-line treatments.

Materials and Methods

Patients

This prospective observational study was started in 2008. A total of 151 patients with histopathologically and clinically diagnosed advanced CRC from January 1, 2008 to June 30, 2014 were enrolled in this project. The following were the criteria for enrolment: (1) patients diagnosed with advanced CRC; (2) histologically confirmed CRC with at least one measurable lesion as defined by the Response Evaluation Criteria in Solid Tumours; (3) patients who received National Comprehensive Cancer Network (NCCN)-guided first-line chemotherapy (FOLFOX or FOLFIRI strategy); (4) patients with completed clinicopathological information and follow-up data; (5) patients have not received previous chemotherapy for the target lesions, however, patients who had received adjuvant/neoadjuvant chemotherapy were enrolled if the interval was more than 12 months after the end of chemotherapy; and (6) sufficient formalin-fixed paraffin-embedded (FFPE) tissues for further study. Prior to primary chemotherapy, tumour samples were acquired during palliative operations or colonoscopy biopsies and embedded in paraffin.

For the training and internal testing sets, 107 metastasis CRC patients treated at Sun Yat-sen University Cancer Center (SYSUCC, Guangzhou, China) were included from January 1, 2008, to June 30, 2014. These 107 patients were randomly divided into training and internal testing sets (67 and 40 patients, respectively). To verify the prognostic or predictive accuracy of the signature, we included another 44 advanced CRC patients as the independent external validation cohort from the Foshan

First People's Hospital (FFPH), Foshan, between December 1, 2008, to December 31, 2013. The same inclusion and exclusion criteria were used. Additionally, to obtain the miRNA expression profiles, 21 frozen tumour and paired adjacent normal mucosa samples from metastatic CRC patients obtained at SYSUCC from January 1, 2003 to December 31, 2005 were used.

The clinical and clinicopathological classification and stage were clarified according to the American Joint Committee on Cancer (AJCC) criteria. Prior to carrying out the research, this project was approved by the Institutional Research Ethics Committee and all the participants signed informed consent forms. All the patients received tumour assessments every six weeks during therapy and at the end of treatment.

Specimen characteristics

The FFPE tissue samples were composed of at least 80% tumour cells. Total RNA was isolated from 151 metastatic CRC samples and 21 paired CRC tumour and adjacent normal tissues as previously described [36]. The purity and concentration of RNA were quantified by using a Nanodrop 2000 spectrophotometer (Thermo Fisher Scientific, USA).

Assay methods

RNA labelling, hybridization and array scanning were carried out as previously described and performed by CapitalBio (Capital-Bio Corp., Beijing, China)[37]. Briefly, total RNA was purified and concentrated using the mirVana™ miRNA Isolation Kit (Ambion, Austin, TX, USA). Purified RNA was labelled with fluorescein Cy3 and hybridization was conducted using an Agilent Human miRNA Microarray (miRBase release 19.0). The fluorescence intensities were converted into digital data and Log2 transformed using Feature Extraction (version 10.7). Differential gene expression was analysed by using GeneSpring software version 12.0 (Agilent, USA). The miRNAs were categorized as significantly differentially expressed if the p value was lower than 0.05. Heat maps were formed using the Cluster 3.0 package software. Microarray data were deposited into the Gene Expression Omnibus (GEO) database of the National Center for Biotechnology Information (NCBI) (accession no. GSE108153).

MiRNA expression was detected by qRT-PCR in the 151 CRC samples and 21 paired CRC and adjacent normal tissues. Total RNA was obtained with TRIzol

reagent (Life Technologies, Carlsbad, California, USA), and 2 μg total RNA was reverse transcribed using the All-in-One™ miRNA First-Strand cDNA Synthesis Kit (GeneCopoeia Inc., USA). The All-in-One™ miRNA qPCR Kit (GeneCopoeia Inc., USA) and Roche Lightcycler 480 instrument (Roche Diagnostics, Basel, Switzerland) were used for qRT-PCR analysis. All experiments were conducted according to the manufacturer's instructions. The results were normalized to U48 expression and analysed using in the 2-Δct method. The primers were synthesized by GeneCopoeia, Inc.

Study design

This prospective observational study was started in 2008. A total of 151 patients with histopathologically and clinically diagnosed advanced CRC were enrolled in this project. For the training and internal testing sets, 107 metastasis CRC patients treated at Sun Yat-sen University Cancer Center (SYSUCC, Guangzhou, China) were included from January 1, 2008, to June 30, 2014. These 107 patients were randomly divided into training and internal testing sets (67 and 40 patients, respectively). To verify the prognostic or predictive accuracy of the signature, we included another 44 advanced CRC patients as the independent external validation cohort from the Foshan First People's Hospital (FFPH), Foshan, between December 1, 2008, to December 31, 2013. The same inclusion and exclusion criteria were used. Follow-up procedure for these patients was finished on July 31, 2016. The median follow-up time was 44.6 month. The clinical end point was PFS, which was defined as the period between the initiation treatment of first-line chemotherapy and the date of disease progression or death.

We performed the miRNA microarray on 21 paired CRC cancer and adjacent normal frozen tissues samples. We identified the differentially expressed miRNAs using the significance analysis of microarrays with a false discovery rate (FDR) <0.05. By combining the RNA expression data from the microarray and follow-up data, we obtained 33 significantly differentially expressed miRNA candidates correlated with PFS by Cox regression analysis ($p<0.1$).

Differential miRNA expression was examined by qRT-PCR in the training set and Cox proportional hazard regression modelling was then performed to analyse the correlation between RNA expression and PFS data in patients. The miRNA signature was validated in the internal and external validation sets. The prognostic or predictive

accuracy of each feature and the miRNA-based signature was investigated by time-dependent receiver operating characteristic (ROC) analysis, which was measured by the area under the curve.

Post hoc statistical power analysis was used to assess adequacy of research design. In the training cohort, the post-hoc statistical power analysis showed an adequate power of 88.3%.

Statistical analysis methods

To determine the miRNAs most predictive of a high/low recurrence risk signature, we utilized the LASSO bagging strategy. At the data preparation step, we first obtained 33 candidate markers from the differential expression and uni-variable Cox regression analysis described above, and we then examined their RNA abundance in a training data set of 67 cancer samples using RT-PCR. Since the relative RT-PCR values of miRNA in each sample were too small to manage, we applied logarithmic transformation (2 bases) on 10,000 multiplied matrices to normalize the values into a readable range. As a result, a matrix of 33 variables and 67 data points (samples) was built for further LASSO bagging analysis, the procedure for which is described as follows: 1) The data points were resampled 1,000 times with replacement to generate 1,000 training matrices. 2) For each matrix with recurrent survival outcomes, we performed LASSO Cox regression analysis using 10-fold cross validation. The tuning parameter λ was selected by 1-SE (standard error), and we finally obtained a list of variables that had beta-coefficients different than zero in LASSO. 3) All variable lists obtained in each resample were combined to calculate the resample model inclusion proportion (RMIP) for each miRNA (also explained by an observed frequency in 1,000 resamples). 4) The RMIP was used as the weight of each variable, and the top 2 miRNAs were selected as candidate markers for building the signature. All running analysis scripts were programmed with R (v3.3.1), and LASSO was adopted from the glmnet package (2.0-10).

Student's *t* test was used to compare continuous variables between paired tumour and adjacent normal tissues. For survival analyses, the curves were plotted with the Kaplan-Meier method, and the different groups were compared using the log-rank test. Adjusted hazard ratios (HRs) with 95% confidence intervals (CI) were calculated using Cox proportional hazards modelling. The median value of the risk score in the training set was taken as the cut-off value. All statistical tests were two-sided and

considered significant at two-sided p values <0.05, unless specifically stated. All the data analyses above were performed using SPSS version 22.0 statistical software or R3.1.2 (R Foundation for Statistical Computing) software.

Results

Data

Clinicopathological characteristics of patients

A total of 151 patients with histopathologically and clinically diagnosed advanced CRC were enrolled in the present study according to the selection criteria. The patients from SYSUCC were included and randomly assigned to the training set (67 patients) and internal testing set (40 patients), and another 44 patients from FPHFS were recruited as the external validation set. The clinicopathological characteristics of the patients in each set are shown in Table 1.

Building a two-miRNA-based prognostic classifier with an integrated marker selection approach and Cox regression

Twenty-one pairs of tumour and adjacent normal tissues from advanced CRC patients were used in the microarray analysis. To obtain the most significant miRNAs for classifying high- and low-risk progressive patients, we applied an integrated marker selection method, consisting of differential expression analysis, uni-variable Cox regression analysis and LASSO bagging variable selection. The flowchart of microRNA filtration is illustrated in Supplementary Fig. S1. First, using the microarray data matrix, differential expression analysis was performed with the limma package between tumour and normal tissues [24]. A total of 178 upregulated miRNAs and 231 down-regulated miRNAs were identified in the tumour samples. Then, with the same data set, Cox regression analyses with a Wald threshold of $p<0.1$ were run individually to screen the progression-related markers. A total of 33 miRNAs were identified as differentially expressed between the tumour and adjacent normal samples and were correlated with the patients' PFS ($p<0.1$, Kaplan-Meier method). Hierarchical clustering showed that the 21 pairs of tumour and adjacent normal tissues were separated into two discrete groups (Fig. 1A). The expression of these 33 miRNAs in tumour and adjacent normal tissues was validated by qRT-PCR (Suppl. Fig. S2), confirming the differential expression of these miRNAs between tumour and adjacent normal tissues.

To further narrow down a miRNA-based signature for patients with advanced CRC, we sequentially applied a LASSO bagging method on the qRT-PCR dataset from the training set. Briefly, the LASSO method is commonly used for regression with high-dimensional predictors, and its extended model has been widely applied to the Cox proportional hazard regression model for the analysis of high-dimensional survival data [25]. For LASSO bagging, bootstrap samples of the data (sampling with replacement with a sample size equal to that of the original data) were used to perform the entire LASSO Cox procedure, including optimal tuning parameter selection (also called λ) and variable selection steps. In the present analysis, we obtained 1,000 resamples and used different optimal λ parameters selected via 1-SE (standard error) criteria for each resample. We then calculated the RMIP for each miRNA. The RMIP is a measure of how likely a given miRNA is selected by the LASSO procedure if the dataset is perturbed. We observed continuing sharp decreases of RMIP until the third miRNA miR-5571-5p as ordered by the RMIP of each miRNA. As a result, two miRNAs, miR-125b-2-3p and miR-933, were selected for building a prognostic classifier (Fig. 1B). Additionally, a two-variable Cox regression analysis was performed on the same data to determine the coefficients of the two miRNAs, which were -0.259 for miR-125b-2-3p and 0.092 for miR-933.

Using a two-variable Cox regression model, we obtained the risk score for each patient (risk score= 0.092×miR-933 – 0.259× miR-125b-2-3p). Based on the signature, the risk score for each patient in the training cohort was determined. The median value of the risk score in the training set was taken as the cut-off value (0.022), and all the patients were then assigned to low- or high-risk groups based on their risk scores.

Analysis and presentation

Prognostic value of the miRNA signature

To examine whether the signature was associated with PFS, we applied multivariate Cox proportional hazards regression analyses in the training, internal testing and external validation sets (Table 2). The patients with high risk scores generally displayed worse PFS than those with lower risk scores in the training set; the median PFS was 12.00 months (95% CI 9.89-14.11 m) for the low-risk group and 7.40 months (95% CI 6.56-8.24 m) for the high-risk group (p<0.001). To confirm the prognostic accuracy of the two-miRNA-based signature, we conducted analyses using

the internal testing and external validation sets. Similar results were observed in these two sets. In the internal testing set, the median PFS was 9.90 months (95% CI 6.89-12.91 m) for the low-risk group and 5.10 months (95% CI 3.57-6.63 m) for the high-risk group ($p<0.001$). In the external validation set, the median PFS was 9.90 (95% CI 7.33-12.47 m) for the low-risk group and 6.40 months (95% CI 4.87-7.93 m) for the high-risk group ($p=0.002$). For all the patients, the median PFS in the low- and high-risk groups was 11.30 (95% CI 9.36-13.24 m) and 7.20 months (95% CI 6.69-7.81 m), respectively ($p<0.001$) (Fig. 2A). The progressive rate was 13.33% (4/30) for the low-risk group and 24.32% (9/37) for the high-risk one in the training set ($p<0.001$); 12.00% (3/25) for the low-risk group and 40.00% (6/15) for the high-risk group in the internal testing set ($p<0.001$); and 17.86% (5/28) vs. 43.75% (7/16), respectively, in the external validation set ($p<0.001$). For all patients, the progressive rate was 14.63% (12/82) for the low-risk group and 31.88% (22/69) for the high-risk one, respectively ($p<0.001$; Fig.2B).

To further assess the prognostic value of the two-miRNA signature on risk score prediction, we calculated the time-dependent receiver operating characteristic (ROC) in the three cohorts respectively. The two-miRNA-based signature showed significantly higher prognostic value than any other single clinicopathological risk factor ($p<0.05$) (Fig. 3).

Predictive value of the signature for chemotherapy sensitivity

We used multivariate Cox proportional hazards regression analysis to test the predictive value of the signature for first-line chemotherapy sensitivity in advanced CRC cases (Table 3). The interactions between the risk status and patient response to chemotherapy were analysed. The interactions between these were dramatically significant in the training set (HR 3.644; 95% CI 1.528-8.690; $p=0.004$), in the internal testing set (HR 8.426; 95% CI 2.592-27.390; $p<0.0001$), and in the external validation set (HR 2.653; 95% CI 1.031-6.829; $p=0.043$). These data suggested that this two-miRNA-based signature might be valuable for predicting the benefit of standard first-line chemotherapy in advanced CRC.

Discussion

Traditional clinicopathologic characteristics, such as gender, age, tumour size, tumour grade, and lymph node status, are rarely associated with the clinical response to chemotherapy of advanced CRC patients. However, the prediction of clinical

response in metastatic CRC is vital for guiding therapeutic decisions. In the present study, we developed and verified a novel two-miRNA-based classifier to predict the disease progression and the benefit of receiving standard first-line chemotherapy in advanced CRC. Our results showed that this classifier could effectively assign advanced CRC patients into high- or low-risk groups with significant differences in PFS and that patients categorized as high risk based on the signature had worse survival outcomes after chemotherapy. Further use of this classifier might help identify advanced CRC patients who are most likely to benefit from first-line chemotherapy. Thus, the present miRNA signature for advanced CRC patients is a valuable prognostic and predictive tool, indicating that patients who are classified as low risk based on their risk scores have much lower chance of disease progression and have better responses to chemotherapy.

There were plenty of miRNAs which were identified differentially expressed in CRC compared with normal tissues. Among these miRNAs, two miRNAs enrolled in the signature, miR-125b and miR-933, have previously been reported as playing potential roles in cancer. MiR-125b acts as either an oncogene or tumour suppressor and shows heterogenic expression in different carcinomas. For example, miR-125b is down-regulated in ovarian, bladder and breast cancer, which suggests a tumour suppressor function [26, 27, 28]. In contrast, miR-125b is upregulated in leukaemia and prostate cancer, where this molecule seems to act as an oncogene [29, 30]. These contradictory findings also occurred in CRC. On the one hand, downregulation of miR-125b was associated with lymph node metastasis in CRC, as shown by significantly increased cell invasion, migration, and MMP activity [31]. On the other hand, high levels of miR-125b expression in CRC were associated with poor survival [32] . In the present study, miR-125b-2-3p was down-regulated in CRC tumour tissues, and the expression of this molecule was positively correlated with PFS, which was consistent with the previous metastatic CRC study [31].

Moreover, our data showed that miR-125b-2-3p affected CRC cell sensitivity to chemotherapeutic therapy. The effects of miR-125b-2-3p knockdown were more notable than those of its overexpression in CRC cells since the expression of miR-125b-2-3p was high in these two CRC cell lines. However, the miR-933 knockdown or overexpression cells had no significant change in their response to the anticancer drugs. Furthermore, the Cox regression analysis showed that there was no significant association between the expression of miR-933 and CRC patients' PFS,

which might mean that the miR-933 is not an independent prognostic and predictive biomarker in CRC.

The function and mechanism of microRNA-933 in CRC tumourigenesis, progression, and response to chemotherapy has not been thoroughly investigated. However, several studies exploring single-nucleotide polymorphisms in miR-933 have shown that this molecule is associated with susceptibility to several types of human cancers [33, 34, 35], although further mechanistic exploration is needed.

However, our study was a prospective observational study and not a clinical trial; therefore, there may be some inherent biases of such a study format. Moreover, the patients enrolled in the present study were all Chinese individuals whose clinical characteristics might be different from those of other ethnic groups; thus, the generalizability of this study might be limited. Clearly, an open-label, multicentre, randomized clinical trial is needed to further validate this novel signature. Furthermore, the mechanism for how miR-125b-2-3p affects CRC sensitivity to chemotherapy warrants further exploration.

In summary, the present study shows that the two-miRNA signature can successfully classify advanced CRC patients into groups with high and low risks of tumour progression. In addition, this signature might be a reliable prognostic and predictive tool for tumour progression in patients with advanced CRC and might be able to predict the benefit of receiving standard first-line chemotherapy in CRC.
